# Reproductive nanotechnology: tretinoin-loaded lipid-core nanocapsulesand *in vitro *embryos production

**DOI:** 10.1186/1753-6561-8-S4-P168

**Published:** 2014-10-01

**Authors:** Caroline Lucas, Mariana Remião, Eliza Rossi, Priscila De Leon, Aline Ourique, William Domingues, Cristina Haas, Vinicius Campos, Ruy Beck, Fabiana Seixas, Tiago Collares

**Affiliations:** 1 Universidade Federal de Pelotas, Pelotas, RS, Brazil; 2 Universidade Federal do Rio Grande do Sul, Porto Alegro, AM, Brazil

## Background

The improvement of *in vitro *maturation (IVM) protocols through the supplementation with different molecules has become an alternative to increase the culture medium efficiency. Tretinoin (TTN, all-*trans *retinoic acid, ATRA), is an active metabolite of vitamin A [1], that mediates cell proliferation, cell differentiation, and embryonic development process. In *in vitro *production embryos systems, TTN acts improving cytoplasmic maturation process in oocytes, developmental competence in early embryos, and quality in blastocysts [2]. Studies have been demonstrated the presence of α, β and γ subtypes of retinoic acid receptors (RARα, RARβ, RAR_γ_) for TTN in oocyte, hatched blastocysts, and cumulus cells [3]. This molecule can also be used for treatment of skin disorders and for anti-tumor treatment, so researchers have been associated TTN with polymeric nanoparticles to protect it from degradation and to improve its chemical stability and efficacy [4]. The aim of present study was to test the concentration-dependent effect of supplementation of free tretinoin (TTN) and tretinoin-loaded lipid-core nanocapsules (TTN-LNC) in bovine *in vitro *maturation media, and its influence in reactive oxygen species (ROS) production in two-to four-cell stage embryos. In conclusion, tretinoin- loaded lipid-core nanocapsules added in *in vitro *maturation media highly protects embryos at early stage of development against oxidative stress.

## Methods

The experimental groups were established, and cumulus-oocyte complexes (COCs) were matured in oocyte *in vitro *maturation medium supplemented with 0.25, 0.5 and 1 μM of TTN-LNC or TTN. Control groups of COCs matured without treatment and treated only with blank lipid-core-nanocapsules (LNC) were also examined. The oocytes were *in vitro *fertilized in order to evaluate the ROS levels in embryos produced by the different treatments. The ROS formation was evaluated in two-to four-cell stage embryos as previously described [5] with some modifications.

## Results and conclusions

ROS production was lower in embryos derived from oocytes matured in the presence of TTN or TTN-LNC. Both treatments TTN and TTN-LCNC protect the cell more effectively against oxidative damage reducing the ROS production. A significant reduction (p <0.05) in ROS production was detected in the presence of TTN-LCNC compared with controls. There was no difference between the concentrations in TTN and TTN-LNC groups. Tretinoin-loaded lipid-core nanocapsules offers an increased protection against oxidative stress in embryos produced *in vitro*. The studies at the molecular level using oocyte competence markers are alternatives to clarify the function of lipid-core nanocapsules in *in vitro *maturation and in embryonic development.
